# Influence of Feeding Weaned Piglets with *Laminaria digitata* on the Quality and Nutritional Value of Meat

**DOI:** 10.3390/foods11071024

**Published:** 2022-03-31

**Authors:** David Miguel Ribeiro, Cristina M. Alfaia, José M. Pestana, Daniela F. P. Carvalho, Mónica Costa, Cátia F. Martins, José P. C. Lemos, Miguel Mourato, Sandra Gueifão, Inês Delgado, Patrícia Carvalho, Diogo Coelho, Inês Coelho, João P. B. Freire, André M. Almeida, José A. M. Prates

**Affiliations:** 1 CIISA—Centre for Interdisciplinary Research in Animal Health, Faculdade de Medicina Veterinária, Universidade de Lisboa, 1300-477 Lisboa, Portugal; davidribeiro@isa.ulisboa.pt (D.M.R.); cpmateus@fmv.ulisboa.pt (C.M.A.); jpestana@fmv.ulisboa.pt (J.M.P.); monicacosta@fmv.ulisboa.pt (M.C.); catiamartins@isa.ulisboa.pt (C.F.M.); jpclemos@fmv.ulisboa.pt (J.P.C.L.); patysofy@gmail.com (P.C.); diogocoelho@fmv.ulisboa.pt (D.C.); 2 LEAF—Linking Landscape, Environment, Agriculture and Food, Instituto Superior de Agronomia, Universidade de Lisboa, 1349-017 Lisboa, Portugal; danielacarvalho@isa.ulisboa.pt (D.F.P.C.); mmourato@isa.ulisboa.pt (M.M.); jpfreire@isa.ulisboa.pt (J.P.B.F.); aalmeida@isa.ulisboa.pt (A.M.A.); 3 INSA—Departamento de Alimentação e Nutrição, Instituto Nacional de Saúde Doutor Ricardo Jorge, Avenida Padre Cruz, 1649-016 Lisboa, Portugal; sandra.gueifao@insa.min-saude.pt (S.G.); ines.delgado@insa.min-saude.pt (I.D.); ines.coelho@insa.min-saude.pt (I.C.)

**Keywords:** *Laminaria digitata*, CAZyme, piglets, meat quality

## Abstract

*Laminaria digitata* is a novel feedstuff that can be used in pig diets to replace conventional feedstuffs. However, its resilient cell wall can prevent the monogastric digestive system from accessing intracellular nutrients. Carbohydrate-active enzyme (CAZyme) supplementation is a putative solution for this problem, degrading the cell wall during digestion. The objective of this work was to evaluate the effect of 10% *L. digitata* feed inclusion and CAZyme supplementation on the meat quality and nutritional value of weaned piglets. Forty weaned piglets were randomly allocated to four experimental groups (*n* = 10): control, LA (10% *L. digitata*, replacing the control diet), LAR (LA + CAZyme (0.005% Rovabio^®^ Excel AP)) and LAL (LA + CAZyme (0.01% alginate lyase)) and the trial lasted for two weeks. The diets had no effect on any zootechnical parameters measured (*p* > 0.05) and meat quality traits, except for the pH measured 24 h post-mortem, which was higher in LAL compared to LA (*p* = 0.016). Piglets fed with seaweed had a significantly lower *n*-6/*n*-3 PUFA ratio compared to control, to which the higher accumulation of C20:5*n*-3 (*p* = 0.001) and C18:4*n*-3 (*p* < 0.0001) contributed. In addition, meat of seaweed-fed piglets was enriched with bromine (Br, *p* < 0.001) and iodine (I, *p* < 0.001) and depicted a higher oxidative stability. This study demonstrates that the nutritional value of piglets’ meat could be improved by the dietary incorporation of *L. digitata*, regardless of CAZyme supplementation, without negatively affecting growth performance in the post-weaning stage.

## 1. Introduction

The human population is estimated to increase above 9 billion people by 2050 [1]. This is expected to increase the demand for animal products, such as pork, thereby increasing the pressure on natural resources such as water and land. It is therefore essential to increase productivity of production systems while maintaining and/or improving environmental sustainability. Feedstuffs commonly used in pig diets, such as soybean meal, have a major environmental impact in both production and transport [2]. In Europe, this impact is worsened by dependency on imports from countries such as the USA and Brazil [3]. Hence, alternative feeds are necessary to reduce Europe’s dependency on these feedstuffs whilst maintaining environmental and economical sustainability. In recent years, researchers have devoted much attention to alternative feedstuffs whose use also reduces food–feed–fuel competition, such as food industry by-products [4], insects [5], microalgae [6,7], and macroalgae [8,9].

Macroalgae (or seaweeds) are a diverse group of multicellular algae with three main categories: *Phaeophyceae* (brown algae), *Rhodophyceae* (red algae), and *Chlorophyceae* (green algae) [10]. Their nutritional composition is highly variable, depending on factors such as species, production/harvesting location, post-harvesting treatment, etc. Green and red algae can have levels of crude protein comparable to those of soybean meal (42–44% on a dry matter–DM-basis), whereas their crude fat content is generally low, below 7% on a DM basis [11]. They also have several bioactive components: *n*-3 polyunsaturated fatty acids (*n*-3 PUFA), including eicosapentaenoic acid (EPA), iodine (I), and polysaccharides such as laminarin [12]. *Laminaria digitata* is a brown seaweed whose laminarin and fucoidan extracts have been widely reported to improve growth and gut health of weaned piglets, as well as meat quality of finishing pigs [13,14,15,16]. To our knowledge, the use of the whole biomass of this seaweed as a feed ingredient (above 3% dietary incorporation) has not been reported. This is likely due to the high abundance of recalcitrant polysaccharides, indigestible by monogastric endogenous enzymes. To take advantage of these nutritional properties, the feed supplementation with carbohydrate-active enzymes (CAZymes) is a viable approach. CAZymes act upon glyosidic bonds of polysaccharides [17]. The commercially available enzyme mixture Rovabio^®^ Excel AP was designed for use in cereal-based diets to degrade non-starch polysaccharides and has been used in microalgae-containing diets [6,7,18]. Alginate lyase, an alginate-degrading enzyme, has been reported to degrade the cell walls of *L. digitata* in vitro [19], being a putative candidate for in vivo studies.

Weaning is a critical stage in pig production, where piglets endure social, nutritional, and environmental-related stress [20]. Antibiotics were intensively used in the past to deal with post-weaning stress, until their use for such purpose was banned in the European Union due to public health concerns. The prebiotic properties and components of seaweeds could help mitigate this issue. For instance, authors have reported that laminarin extracted from *L. digitata* improved the microbiome of weaned piglets, without detrimental effects on growth performance [13], while another study reports growth improvement by feeding piglets with a laminarin extract [14]. Other authors have reported that feeding pigs with laminarin and fucoidan extracts reduced saturated fatty acids and lowered lipid oxidation in meat [15]. This is particularly interesting in the Southern European context, given that there is a tradition of spit-roast piglet consumption in countries such as Portugal (*Leitão de Negrais*).

Following the discussion above, the objective of this work was to evaluate the effect of 10% dietary *L. digitata* and CAZyme supplementation on the quality and nutritional value of weaned piglets’ meat.

## 2. Materials and Methods

### 2.1. Animals and Experimental Diets

The animal trial was conducted at the Animal Production Section of ISA (University of Lisbon, Portugal). It was approved by ISA’s Ethics Commission and accepted by the National Veterinary Authority (ref. 0421/000/000/2020) following current legislation of the European Union (2010/63/EU Directive). Forty male piglets (Large White × Duroc), 35-day old, with 10.49 ± 0.62 kg (mean ± SD) body weight were bought from a commercial farm, where they had been weaned at 28 days of age. They were housed individually in metabolic crates, equipped with individual heating lamps and nipple drinkers. The piglets were then randomly allocated to each of the four experimental groups (*n* = 10): control (maize, wheat, and soybean meal-based diet), LA (10% *L. digitata*, replacing the control diet), LAR (LA + CAZyme-0.005% Rovabio^®^ Excel AP from Adisseo (Antony, France)) and LAL (LA + CAZyme-0.01% pre-selected alginate lyase as described by Costa et al. [19]). Experimental diet composition is presented in Table S1. The seaweed was wild caught, bought from Aleor (Lézardrieux, France), and used as supplied (dried powder, <250 µm). The seaweed had 4.85% and 1.31% (on a dry matter basis) of crude protein and crude fat, respectively. Diets were formulated to be isocaloric and isonitrogenous.

### 2.2. Diet Composition Analysis

#### 2.2.1. Proximal Analysis

Procedures for proximate analysis of diets were previously described [6]. Briefly, dry matter (DM) was assessed by drying diets at 103 °C overnight. Ash was determined by calcinating the dried sample at 500 °C in a muffle furnace overnight. Crude protein was measured using the Kjeldahl method, using 6.25 as conversion factor. Crude fat was determined by hydrolysis followed by automatic Soxhlet extraction with petroleum ether (Gerhardt Analytical Systems, Königswinter, Germany). Crude energy was analysed by adiabatic calorimeter (Parr 1261; Parr Instrument Company, Moline, IL, USA). The AOAC guidelines were followed [21].

#### 2.2.2. Fatty Acid Composition

Fatty acid composition of diets was assessed by one-step extraction and acid transesterification [22]. Afterwards, fatty acid methyl esters (FAME) derivatives were separated, identified, and quantified through gas chromatography (GC), following previously reported conditions [22]. The identification of fatty acids was done using the reference standard (FAME mixture of 37 compounds, Supelco Inc., Bellefonte, PA, USA). The internal standard was nonadecanoic acid (C19:0) methyl ester. Fatty acids were quantified as percentage of total fatty acids.

#### 2.2.3. Pigment Profiling

Diterpene profile and β-carotene content of diets were evaluated based on direct saponification and single extraction with n-hexane and then analysed by HPLC as carried out by Prates et al. [23]. Pigments in diets were determined according to Teimouri et al. [24]. Approximately 0.5 g of diets were incubated with acetone in the dark at room temperature during 12 h under agitation. After extraction, samples were centrifuged and analysed by UV-VIS spectrophotometry (Ultrospec 3100; Amersham Biosciences, Little Chalfont, UK). The amount of pigments was calculated using the Hynstova et al. [25] equations as follows: clorophyll-*a* (Ca) = 11.24 A662 − 2.04 A645; clorophyll-*b* (Cb) = 20.13 A645 − 4.19 A662; total chlorophylls (Ca + Cb) = 7.05 A662 + 18.09 A645; total carotenoids (Cx + c) = (1000 A470 − 1.90 Ca − 63.14 Cb)/214; and total chlorophylls and total carotenoids (Ca + Cb) + (Cx + c).

#### 2.2.4. Mineral Profiling

The mineral profile of experimental diets was analysed as described by Ribeiro et al. [26]. Diets were incubated in a ventilated chamber with concentrated nitric acid plus hydrochloric acid, during 16 h, followed by the addition of hydrogen peroxide and heated using a digestion plate (DigiPREP MS, SCP Science, Baie-D’Urfe, QC, Canada). Then, diets were diluted with distilled water, filtered, and analysed by Inductively Coupled Plasma–Optical Emission Spectrometry (ICP-OES, iCAP 7200 duo Thermo Scientific, Waltham, MA, USA). The analysis of I and bromine (Br) was performed by Inductively Coupled Plasma Mass Spectrometer (ICP-MS) (Thermo X series II, Thermo Fisher Scientific, Waltham, MA, USA), according to Delgado et al. [27]. Briefly, tetramethylammonium hydroxide (TMAH) solution (25%, *v*/*v*) and ultra-pure water (Milli-Q Element system, Millipore Corporation, Saint-Quentin, France) were added to samples followed by extraction, in triplicate, using a Heating Graphite Block System (DigiPREP MS, SCP Science, Baie-D’Urfe, QC, Canada) at 90 °C during 3 h.

The detailed chemical composition of the experimental diets is presented in Table 1.

### 2.3. Growth Performance and Slaughter of Piglets

After 5 days of adaptation to diets and environmental conditions, the trial lasted for two weeks, and piglets had free access to water. Feed refusals were recorded daily. Piglets were weighed at the beginning and end of each week to calculate growth performance parameters. At the end of trial, all piglets were slaughtered by electrical stunning and exsanguination, following commercial practices. The *longissimus lumborum* (LL) muscle was removed from each carcass between the third and fifth lumbar vertebrae. Meanwhile, LL muscle from the right carcass was used for meat quality traits and sensory analyses, and LL muscle from the left carcass side was minced, vacuum packed, and stored at −20 °C for biochemical analysis.

### 2.4. Measurement of Meat Quality Traits and Sensory Analysis

Meat pH was measured, in triplicate, at different positions, on LL muscle at 24 h post-mortem using a pH meter (Hanna Instruments, Woonsocket, RI, USA) equipped with an insertion glass electrode. At 24 h post-mortem, meat colour parameters lightness (L*), redness (a*), and yellowness (b*) were measured three times on the exposed (after blooming for 60 min at 4 °C) cut surface of the LL muscle by using a CR-300 Minolta colorimeter (Tokyo, Japan). Chroma (C*, colour intensity also known as saturation index) was calculated as (a*^2^ + b*^2^)^1/2^. Hue angle (H*) was calculated as tan^−1^ (b*/a*) × 57.29, expressed in degrees. To determine shear force and cooking loss, meat samples were thawed at 4 °C for 24 h and cooked in a water bath programmed at 80 °C with a meat internal temperature of 78 °C monitored by a thermocouple (Lufft C120; Lufft, München, Germany). Shear force was determined in meat samples, along the direction of the muscle fibres with a 1 cm^2^ cross-section, using a Warner-Bratzler blade coupled to a texture analyser (TA-XT Plus texture analyser; Stable Micro Systems, Surrey, UK). Cooking loss was calculated as a percentage of weight before and after cooking.

Twelve panellists, specifically trained in five panel sessions with 8 random meat samples per session, were selected in accordance with Cross et al. [28]. The sensory descriptors were tenderness, juiciness, flavour, off-flavour, and overall acceptability. A graduated scale from 1 to 8 was used to quantify these descriptors, where 1 represents the lowest score and 8 the highest score. For off-flavour, the scale used was from 0 (absence) to 8 (maximum).

### 2.5. Intramuscular Fat Content and Fatty Acid Composition Determination

Lipid fraction from lyophilized LL muscle samples was extracted by combining the traditional Folch method [29] with that applied by Carlson [30], in which dichloromethane-methanol (2:1, *v*/*v*) was used as binary solvent mixture. After solvent evaporation, intramuscular fat was measured gravimetrically by weighing the residue. To profile meat fatty acids, lipid extracts were transmethylated in combined alkaline and acid conditions, according to Raes et al. [31]. FAME were identified and quantified by gas chromatography (HP6890A; Hewlett-Packard, Avondale, PA, USA), as reported by Coelho et al. [18]. As mentioned above for diets, nonadecanoic acid (C19:0) methyl ester was the internal standard and fatty acids are expressed as percentage of total fatty acids.

### 2.6. Total Cholesterol, Diterpene Profile, and Lipid Oxidation Determination

The quantitative analysis of total cholesterol, β-carotene, and vitamin E homologues (tocopherols and tocotrienols) in LL muscle, in duplicate, was performed as described by Prates et al. [23].

The lipid oxidation status of meat was evaluated in terms of thiobarbituric acid reactive substances (TBARS) at days 0 and 8, maintained at 4 °C, according to Grau et al. [32]. TBARS values, in duplicate, were obtained and expressed as mg of malondialdehyde (MDA) per kg of meat.

### 2.7. Mineral Profile Determination

The determination of mineral profile in LL muscle was done following the same procedure as diet samples (for further information see details above, Section 2.2.4).

### 2.8. Statistics

All data were analysed with the GLM procedure of the SAS software (version 9.4; SAS Institute Inc., Cary, NC, USA), except TBARS that were analysed with MIXED procedure of SAS. The dietary treatment was considered as the single effect, and the piglet was the experimental unit. Upon detection of significant effects (*p* < 0.05), least-square means were compared using the PDIFF option, adjusted for the Tukey post hoc test. The statistical models used were Yi = µ + τi + εi, (Proc GLM) and Yi = µ + τi + ω(piglet)i + εi (Proc Mixed). Yi is the response of piglet in treatment i, µ is the global average of the effect, τi is the effect of treatment i, ω(piglet) is the effect of time within piglet and εi is the residual error.

A principal component analysis (PCA) was carried out for the fatty acid profile of LL muscle using the *PCA* and *fviz_pca_biplot* functions of the FactoMineR [33] and factoextra packages (respectively) of the R software (version 3.6.2; R Foundation for Statistical Computing, Vienna, Austria [34]).

## 3. Results

### 3.1. Effect of the Experimental Diets on Zootechnical Parameters

Diets had no statistically significant effect on live weight (*p* > 0.05). Piglets had, on average, 11.6 kg and 16.8 kg of initial and final weight, respectively. The average daily gain, average daily feed intake, and feed conversion ratio were also unaffected, with average 371 g, 645 g, and 1.8, respectively.

### 3.2. Effect of the Experimental Diets on Meat Quality Traits and Sensory Panel Analysis

Table 2 summarizes the effect of experimental diets on meat quality traits and sensory panel analysis in weaned piglet meat. The pH 24 h post-mortem was higher in the LAL compared with the LA treatment (*p* = 0.016). However, experimental diets had no significant effect on colour parameters, cooking loss and shear force (*p* > 0.05). Regarding the sensory panel analysis, there were no significant differences for tenderness, juiciness, flavour, off-flavour, and overall acceptability (*p* > 0.05).

### 3.3. Effect of the Experimental Diets on Intramuscular Fat, Total Cholesterol and Vitamin E Content, and Fatty Acid Composition

The effect of experimental diets on intramuscular fat (IMF), total cholesterol, and fatty acid profile of LL muscle is shown in Table 3. The IMF of LAR piglets tended to be higher than its counterparts (*p* = 0.063), whereas for total cholesterol and α-tocopherol, no statistically significant effect due to experimental diets (*p* > 0.05) was recorded. Among the diterpenes, only α-tocopherol, the major homologue of vitamin E, was detected in LL muscle. Furthermore, chlorophylls and carotenoids (including β-carotene) were not detected in LL muscle, even though they were present in the diets in low amounts.

A PCA analysis for the lipid profile of the LL muscle is depicted in Figure S1. There is not a clear clustering of experimental groups, which is explained by the low number of differences found for individual FA, as indicated by the biplot vectors. The concentration of stearidonic acid (C18:4*n*-3) was significantly higher in *L. digitata* fed groups compared to control (*p* < 0.0001), and eicosapentaenoic acid (EPA, C20:5*n*-3) was 43% higher in LA compared to control (*p* = 0.001). Docosadienoic acid (C22:2*n*-6) tended to be higher in control (*p* = 0.068), whereas docosapentaenoic acid (C22:5*n*-3) and eicosatrienoic acid (C20:3*n*-3) tended to be higher in seaweed diets (*p* = 0.066 and *p* = 0.068, respectively). Experimental diets had no effect on any SFA and monounsaturated fatty acids (MUFA) (*p* > 0.05).

There was no significant effect detected in the PUFA/SFA ratio (*p* > 0.05). However, there was a significant effect (*p* < 0.0001) in the *n*-6/*n*-3 ratio, where control had increased by 68%, 72%, and 78% compared to LA, LAR, and LAL, respectively.

### 3.4. Effect of the Experimental Diets on Meat Oxidative Stability

**Figure 1:**
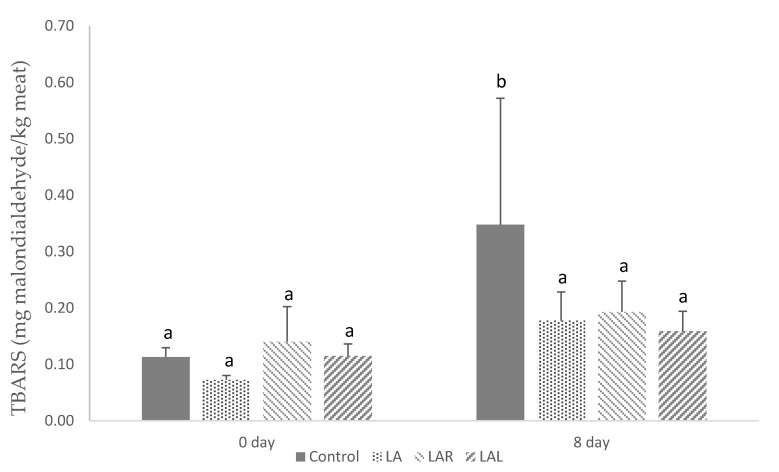
TBARS levels (mg malondialdehyde/kg muscle) after 0 and 8 days under refrigeration in *longissimus lumborum* muscle of piglets fed the experimental diets. Control, LA, LAR, and LAL diets represent corn-soybean meal-based diets containing 0% *L. digitata* (Control), 10% *L. digitata* (LA), 10% *L. digitata* + 0.005% of Rovabio^®^ Excel AP (LAR); and 10% *L. digitata* + 0.01% of alginate lyase recombinant CAZyme (LAL). ^a,b^ Values with different letters are significantly different (*p* < 0.05).

Figure 1 displays the influence of diets on the oxidative stability of piglets’ LL muscle. Data showed no significant effects of lipid oxidation between groups within each time among the experimental diets (*p* > 0.05). However, there was a significant increase of TBARS concentration in the control group between 0 and 8 days (*p* < 0.05), which did not occur in the remaining groups.

### 3.5. Effect of the Experimental Diets on Mineral Profile

The mineral concentration of LL muscle is presented in Table 4. Concerning macrominerals, no significant differences were detected between treatments, albeit having a strong tendency for calcium (Ca, *p* = 0.05) to be higher in LA compared to the remaining groups. On the other hand, Br (*p* < 0.001) and I (*p* < 0.001) were higher in the muscle of piglets fed seaweed diets, which led to a significant increase of total microminerals content when compared with control (*p* < 0.001). Moreover, the heavy metals arsenic, barium, cadmium, chromium, cobalt, nickel, lead, and vanadium were not detected in the muscle as observed in the experimental diets.

## 4. Discussion

This study is, to our knowledge, the first to report inclusion levels of *L. digitata* reaching 10%, as well as the effects of CAZyme supplementation on growth performance and meat quality traits of weaned piglets. We found that diets with the inclusion of the seaweed used in this study had no effect on piglet growth or feed intake. This is coherent with previous studies using lower inclusion levels of seaweeds. Brugger et al. [8] have reported that diets containing up to 5% whole *Laminaria japonica* have no detrimental effect on piglet growth but did improve feed conversion ratio compared to the control diet. Other authors have reported similar results obtained by supplementing piglet diets with fucoidan (250 ppm) extracted from *Ascophyllum nodosum*. Indeed, the authors found no effect on piglet growth, but an improvement of feed conversion ratio in supplemented piglets compared to the control group [13]. The reason why we did not find such an effect in the present study could be related to the different seaweed species. Nevertheless, our study demonstrates that there is no detrimental effect in growth performance by feeding piglets with up to 10% whole biomass *L. digitata*, regardless of enzymatic supplementation.

Regarding meat quality traits, there were no differences in traits including meat colour, cooking loss, or any score from the sensory panel evaluation. However, there was a significant increase in 24 h post-mortem pH of LAL meat compared to LA. Meat pH is a determinant factor in the development of pork quality attributes [35], such as tenderness, juiciness, and flavour [36]. The pH of meat is lowered post-mortem, during the transition muscle to meat where, under anaerobic conditions, glycogen metabolization and ATP hydrolysis accumulate lactate and H^+^, respectively [37]. The normal range of ultimate pH is between 5.5 and 5.7, where desirable meat traits are developed. The values reported in this study are within this range, pointing towards absence of undesirable meat development such as pale-soft and exudative meat (PSE, pH < 5.4). Authors have reported no effects of feeding pigs with *Macrocystis pyrifera* (up to 4%), a brown seaweed, on meat pH of finishing pigs [38]. Another study has also reported no effect of feeding pigs with a *L. digitata* polysaccharide extract on pork patties pH [39]. Therefore, the reason why we found a higher pH in LAL compared to LA could be the higher dietary inclusion of *L. digitata* and enzyme supplementation. This influenced the muscle glycogen content and, ultimately, the rate of pH decline, without detrimental effects on meat sensory properties.

The fatty acid profile has been significantly changed in the meat of seaweed-fed piglets compared to control. Regarding individual fatty acids, control piglets had significantly lower levels of C18:4*n*-3 compared to the remaining groups, whereas LA accumulated significantly more C20:5*n*-3 when compared to controls. C18:4*n*-3 was 21 times more concentrated in *L. digitata* diets whereas C20:5*n*-3 was not detected in the control diet, which explains these differences as being due to their dietary availability. In fact, seaweeds have been reported as having low amounts of fat compared to other feedstuffs, but the fatty acid profile is generally rich in *n*-3 PUFA [11,12], which have a beneficial effect on health. Enrichment of pig meat with *n*-3 PUFA through dietary manipulation has been achieved with other nutrient sources, including microalgae [6] or linseed [40]. Regarding seaweeds, there are few studies that report the fatty acid profile of meat from animals fed with these novel feedstuffs. Moroney et al. [15] have reported that dietary laminarin extracted from *L. digitata* reduced SFA in the *longissimus dorsi* (LD) muscle of pigs, without an effect in *n*-3 PUFA. This suggests that the enrichment reported in the present study is independent of the seaweed’s bioactive polysaccharides. Indeed, the higher dietary availability of *n*-3 PUFA in *L. digitata* diets ultimately contributed to a significantly lower *n*-6/*n*-3 ratio, which favours the nutritional value of the meat. Researchers have advised that this ratio should be kept to a maximum of 4 in human diets [41], in order to reduce the incidence of cardiovascular diseases (CVD). Importantly, the long-chain *n*-3 PUFA, EPA, contributed to these results. This FA has been reported as a contributor for reduced incidence of cancer, obesity, and diabetes, in addition to CVD [42]. Finally, this was achieved without compromising the oxidative stability of meat, which has been reported in *n*-3 PUFA enriched pork [6], due to the propensity of these long chain PUFA to be oxidised.

We found that control piglets had significantly higher TBARS in meat after 8 days of refrigeration, compared to the remaining groups. This has happened despite an increase of *n*-3 PUFA accumulation in seaweed-fed piglets. It could be explained by an increased accumulation of pigments with antioxidant activity, but it was not the case because these were not detected in the meat (data not shown). Therefore, this reduced oxidation in *L. digitata* treatments could occur due to the prebiotic activity of antioxidative polysaccharides such as laminarin and fucoidan. Authors have found that feeding pigs with *L. digitata* extracts containing them reduces meat oxidation [15]. To our knowledge, the precise action of this mechanism remains to be elucidated [12].

Finally, seaweed diets promoted an accumulation of Br and I in the LL of piglets leading to an increase of total sum of microminerals in relation to control. These results are explained by the high amount of both minerals in *L. digitata*, which is within the range of values already reported [11], and thus in the respective experimental diets. Accordingly, previous studies described a significant accumulation of I in the muscle of pigs fed brown seaweeds. For instance, feeding piglets with 2% *Ascophylum nodosum* led to an increase of 36% of I concentration in the LD muscle in comparison with the control [43]. In addition, supplementing pig diets with up to 0.186% *L. digitata* was shown to enrich *gluteus maximus* muscle in I by 45% when compared with the control [44]. The organic I from *L. digitata* is readily metabolised and deposited in piglet muscle [45,46]. To our knowledge, there is no available data concerning the accumulation of Br in the meat of seaweed-fed pigs. The Br:I ratio reached 2.66 in LAR. This ratio should be kept low to prevent goitrogenic effects derived from excess bromine [47]. However, it is known that Br is also an essential nutrient due to its requirements for collagen IV formation [48]. Thus, we demonstrated that feeding piglets with *L. digitata* provides an important source of microminerals, which are of paramount importance to maintain physiological functions at the critical post-weaning stage [49].

## 5. Conclusions

The dietary incorporation of *L. digitata* in piglet diets had no detrimental effect on either growth performance or meat quality. CAZyme supplementation was not necessary to improve several meat nutritional variables, including fatty acid composition, mineral profile, and oxidative stability. This contributes to the production of healthier meat without the need for feed supplementation, promoting the intake of *n*-3 PUFA, particularly EPA, without recurring to unsustainable sources such as fish oil. Ultimately, this study supports the feasibility of reducing the incorporation of conventional feedstuffs in piglet diets using seaweeds. However, further research is necessary, namely, to evaluate the digestibility of *L. digitata* at these incorporation levels and its impact in piglet metabolism.

## Figures and Tables

**Table 1:** Chemical composition of experimental diets.

	Dietary Treatments			
	Control	LA	LAR	LAL
Proximate composition (% dry matter)				
Dry matter	89.4	89.6	89.7	89.5
Crude protein	18.5	17.0	17.0	17.4
Crude fat	3.9	4.0	4.6	4.1
Ash	5.9	6.4	6.5	6.3
Crude energy (cal/g DM)	4390.23	4306.12	4287.53	4339.46
Fatty acid composition (% total FA)				
Myristic acid (C14:0)	0.435	0.612	0.502	0.476
Palmitic acid (C16:0)	10.6	11.3	11.0	10.9
Palmitoleic acid (C16:1*c*9)	0.163	0.258	0.254	0.236
Margaric acid (C17:0)	0.074	0.075	0.073	0.071
cis-9 Margaric acid (C17:1*c*9)	0.037	0.044	0.044	0.040
Stearic acid (C18:0)	3.32	3.23	3.26	3.31
Oleic acid (C18:1*c*9)	25.6	25.2	25.3	25.3
Linoleic acid (C18:2*n*-6)	55.8	54.1	54.6	54.8
Linolenic acid C18:3*n*-3	1.33	1.45	1.44	1.48
Stearidonic acid (C18:4*n*-3)	0.009	0.193	0.193	0.193
Arachidic acid (C20:0)	0.311	0.328	0.331	0.334
Arachidonic acid (C20:4*n*-6)	0.007	0.308	0.314	0.278
Eicosapentaenoic acid (C20:5*n*-3)	n.d.	0.382	0.396	0.351
Diterpene profile (μg/g DM)				
α-Tocopherol	59.5	51.8	45.7	45.4
β-Tocopherol	0.978	0.733	0.708	0.830
γ-Tocopherol	2.35	1.59	1.51	1.94
δ-Tocopherol	0.525	0.447	0.448	0.475
γ-Tocotrienol	1.62	1.41	1.31	1.50
Pigments ^1^ (µg/g DM)				
β-Carotene	0.418	1.46	1.44	1.31
Chlorophyll-*a*	0.324	36.2	36.8	36.0
Chlorophyll-*b*	3.791	1.21	0.727	0.714
Total chlorophylls	4.02	35.4	36.6	34.7
Total carotenoids	0.461	12.5	12.0	10.5
Total chlorophylls + carotenoids	4.49	48.0	48.6	45.3
Mineral profile (mg/kg DM)				
Bromine	15.1	83.1	80.8	87.7
Calcium	17,445	16,022	16,675	15,931
Copper	274	244	269	236
Iodine	9.56	652	647	713
Iron	304	226	253	246
Magnesium	1751	2615	2605	2569
Manganese	149	123	123	113
Phosphorous	11,131	6381	6445	6167
Potassium	12,789	15,694	15,651	15,680
Sodium	4542	6647	6462	6321
Sulphur	3094	4787	4726	4550
Zinc	229	254	269	233

Control, LA, LAR, and LAL diets represent corn-soybean meal-based diets containing 0% *L. digitata* (Control), 10% *L. digitata* (LA), 10% *L. digitata* + 0.005% of Rovabio^®^ Excel AP (LAR); and 10% *L. digitata* + 0.01% of alginate lyase recombinant CAZyme (LAL). DM, dry matter; FA, fatty acids; n.d., not detected. ^1^ Chlorophyll-*a*, chlorophyll-*b*, total chlorophylls, and total carotenoids were calculated as described by Hynstova et al. [25].

**Table 2:** pH, colour, cooking loss, shear force and sensory panel analysis in *longissimus lumborum* of piglets fed the experimental diets.

	Dietary Treatments					
	Control	LA	LAR	LAL	SEM	*p*-Value
pH 24 h	5.57 ^ab^	5.53 ^a^	5.63 ^ab^	5.66 ^b^	0.029	0.016
Colour						
L*	48.0	48.9	47.9	46.6	0.670	0.129
a*	7.43	7.32	6.99	7.22	0.279	0.721
b*	0.256	0.587	0.216	0.324	0.280	0.785
C*	7.40	7.38	7.30	7.48	0.287	0.981
H*	2.05	4.24	1.26	1.55	2.229	0.777
Cooking loss (%)	34.7	35.0	34.1	33.2	0.589	0.166
Shear force (kg)	4.02	3.75	4.94	5.05	0.463	0.132
Sensory panel scores						
Tenderness	5.52	5.92	5.72	5.86	0.120	0.084
Juiciness	5.61	5.89	5.76	5.70	0.109	0.324
Flavour	5.60	5.53	5.63	5.57	0.093	0.908
Off-flavour	0.192	0.367	0.242	0.227	0.068	0.285
Overall acceptability	5.62	5.74	5.76	5.79	0.106	0.678

Control, LA, LAR, and LAL diets represent corn-soybean meal-based diets containing 0% *L. digitata* (Control), 10% *L. digitata* (LA), 10% *L. digitata* + 0.005% of Rovabio^®^ Excel AP (LAR); and 10% *L. digitata* + 0.01% of alginate lyase recombinant CAZyme (LAL). SEM, standard error of the mean. ^a,b^ Different superscript letters within a row are significantly different (*p* < 0.05).

**Table 3:** Intramuscular fat, total cholesterol and α-tocopherol contents, and fatty acid (FA) composition (% of total FA) in *longissimus lumborum* muscle of piglets fed the experimental diets.

	Dietary Treatments					
	Control	LA	LAR	LAL	SEM	*p*-Value
Intramuscular fat (g/100 g of muscle)	1.43	1.60	1.88	1.58	0.117	0.063
Total cholesterol (mg/100 g muscle)	35.6	36.8	30.8	33.3	0.038	0.621
α-Tocopherol (µg/100 g)	73.0	86.3	70.2	64.9	6.1	0.103
Fatty acid profile (% of total FA)						
Lauric acid (C12:0)	0.035	0.022	0.038	0.032	0.008	0.503
Myristic acid (C14:0)	0.879	0.803	0.864	0.891	0.069	0.814
Pentadecanoic acid (C15:0)	0.160	0.180	0.181	0.193	0.016	0.546
Palmitic acid (C16:0)	23.3	22.5	23.1	23.7	0.491	0.419
Margaric acid (C17:0)	0.607	0.774	0.721	0.786	0.060	0.151
Stearic acid (C18:0)	13.7	13.8	13.5	14.0	0.443	0.831
Arachidic acid (C20:0)	0.139	0.149	0.156	0.150	0.009	0.589
Behenic acid (C22:0)	0.050	0.106	0.077	0.046	0.023	0.233
Total SFA	38.9	38.4	38.6	39.8	0.669	0.458
Myristoleic acid (C14:1*c*9)	0.006	0.004	0.005	0.010	0.004	0.724
*c*7-Hexadecenoic acid (C16:1*c*7)	0.373	0.376	0.375	0.367	0.014	0.964
Palmitoleic acid (C16:1*c*9)	2.48	2.04	2.44	2.42	0.236	0.528
cis-9 Margaric acid (C17:1*c*9)	0.284	0.299	0.365	0.331	0.035	0.369
Oleic acid (C18:1*c*9)	24.2	21.5	23.4	23.2	1.39	0.463
Vaccenic acid (C18:1*c*11)	3.83	3.68	3.80	3.68	0.173	0.903
Eicosenoic acid (C20:1*n*-9)	0.375	0.346	0.370	0.371	0.023	0.813
Erucic acid (C22:1*n*-9)	0.062	0.117	0.084	0.056	0.025	0.303
Total *cis*-MUFA	31.6	28.0	30.8	30.4	1.59	0.426
Linoleic acid (C18:2*n*-6)	20.6	22.1	20.7	20.5	0.976	0.600
γ-linolenic acid (C18:3*n*-6)	0.060	0.060	0.06	0.054	0.004	0.601
Linolenic acid (C18:3*n*-3)	0.334	0.403	0.399	0.339	0.042	0.505
Stearidonic acid (C18:4*n*-3)	0.028 ^a^	0.115 ^b^	0.120 ^b^	0.096 ^b^	0.012	<0.0001
Eicosadienoic acid (C20:2*n*-6)	0.576	0.611	0.578	0.538	0.030	0.422
γ-homolinolenic acid (C20:3*n*-6)	0.471	0.537	0.494	0.491	0.048	0.794
Arachidonic acid (C20:4*n*-6)	4.41	5.12	4.51	4.55	0.602	0.834
Eicosatrienoic acid (C20:3*n*-3)	0.067	0.099	0.093	0.066	0.011	0.068
Eicosapentaenoic acid (C20:5*n*-3)	0.070 ^a^	0.163 ^b^	0.114 ^ab^	0.118 ^ab^	0.014	0.001
Docosadienoic acid (C22:2*n*-6)	0.036	0.012	0.014	0.012	0.007	0.068
Docosapentaenoic acid (C22:5*n*-3)	0.368	0.606	0.511	0.505	0.061	0.066
Docosahexaenoic acid (C22:6*n*-3)	0.253	0.366	0.315	0.293	0.033	0.127
Total PUFA	27.2	30.2	27.9	27.6	1.67	0.583
Total *n*-3 PUFA	1.12 ^a^	1.75 ^b^	1.55 ^b^	1.42 ^ab^	0.111	0.003
Total *n*-6 PUFA	26.1	28.5	26.4	26.2	1.58	0.673
Other	2.25	3.38	2.65	2.21	0.329	0.059
Ratios of fatty acids						
PUFA/SFA	0.710	0.803	0.730	0.697	0.052	0.478
*n*-6/*n*-3	24.2 ^a^	16.5 ^b^	17.4 ^b^	18.9 ^b^	1.04	<0.0001

Control, LA, LAR, and LAL diets represent corn-soybean meal-based diets containing 0% *L. digitata* (Control), 10% *L. digitata* (LA), 10% *L. digitata* + 0.005% of Rovabio^®^ Excel AP (LAR); and 10% *L. digitata* + 0.01% of alginate lyase recombinant CAZyme (LAL). SEM, standard error of the mean; SFA, saturated fatty acids; MUFA, monounsaturated fatty acids; PUFA, polyunsaturated fatty acids. ^a,b^ Different superscript letters within a row are significantly different (*p* < 0.05).

**Table 4:** Mineral content in *longissimus lumborum* muscle of piglets fed the experimental diets.

	Dietary Treatments					
	Control	LA	LAR	LAL	SEM	*p*-Value
Macrominerals (mg/100 g)						
Calcium	22.8	25.9	22.6	21.8	1.07	0.050
Magnesium	511	508	505	496	7.8	0.533
Potassium	34.4	33.5	33.9	33.5	0.54	0.595
Phosphorous	56.6	57.1	59.8	57.8	1.31	0.367
Sodium	297	293	295	293	8.9	0.986
Sulphur	194	191	190	182	3.7	0.128
Total	1116	1109	1106	1083	15.9	0.503
Microminerals (mg/100 g)						
Bromine	0.108 ^b^	0.430 ^a^	0.473 ^a^	0.491 ^a^	0.0163	<0.001
Copper	0.14	0.12	0.13	0.14	0.006	0.281
Iodine	0.002 ^b^	0.183 ^a^	0.178 ^a^	0.194 ^a^	0.0112	<0.001
Iron	1.00	1.06	0.98	0.96	0.052	0.593
Manganese	0.045	0.039	0.045	0.043	0.0023	0.218
Zinc	1.24	1.42	1.41	1.28	0.066	0.131
Total	2.52 ^b^	3.26 ^a^	3.22 ^a^	3.11 ^a^	0.079	<0.001
Total macro- and microminerals	1118	1112	1109	1086	15.9	0.513

Control, LA, LAR, and LAL diets represent corn-soybean meal-based diets containing 0% *L. digitata* (Control), 10% *L. digitata* (LA), 10% *L. digitata* + 0.005% of Rovabio^®^ Excel AP (LAR); and 10% *L. digitata* + 0.01% of alginate lyase recombinant CAZyme (LAL). SEM, standard error of the mean. ^a,b^ Different superscript letters within a row are significantly different (*p* < 0.05).

## Data Availability

The data presented in this study are available on request from the corresponding author.

## Supplementary Materials

The following are available online at https://www.mdpi.com/article/10.3390/foods11071024/s1, Table S1: Dietary composition of control (maize, wheat and soybean meal-based diet), LA (10% *Laminaria digitata*, replacing the control diet), LAR (LA + CAZyme − 0.005% Rovabio^®^ Excel AP from Adisseo (Antony, France)) and LAL (LA + CAZyme − 0.01% pre-selected alginate lyase). Figure S1: PCA and biplot obtained for the fatty acid profile of *longissimus lumborum* muscle piglets fed with control (ctrl–maize, wheat and soybean meal-based diet), LA (10% *Laminaria digitata*), LAR (10% *L. digitata* + 0.005% of Rovabio^®^ Excel AP) and LAL (10% *L. digitata* + 0.01% of alginate lyase recombinant CAZyme).
