# Supplementary material for: Influence of Feeding Weaned Piglets with Laminaria digitata on the Quality and Nutritional Value of Meat
Source: Foods. 2022 Mar 31;11(7):1024. doi: 10.3390/foods11071024 (PMC8997633; doi:10.3390/foods11071024)
Supplement: Supplementary file 1 [file foods-11-01024-s001.zip › foods-1604164-supplementary/Table S1.pdf]

Table S1 – Dietary composition of control (maize, wheat and soybean meal-based diet), LA (10% *Laminaria digitata*, replacing the control diet), LAR (LA + CAZyme - 0.005% Rovabio® Excel AP from Adisseo (Antony, France)) and LAL (LA + CAZyme - 0.01% pre-selected alginate lyase).

	**Control**	**LA**	**LAR**	**LAL**
**Ingredient composition (g/kg)**				
Wheat	437	393	393	393
Corn	150	135	135	135
Soybean meal 44	250	225	225	225
Sweet whey powder	100	90	90	90
Sunflower oil	30	27	27	27
Laminaria digitata	-	100	100	100
L-Lysine	5.0	4.5	4.5	4.5
DL-Methionine	1.0	0.9	0.9	0.9
L-Threonine	1.0	0.9	0.9	0.9
Calcium carbonate	5.0	4.5	4.5	4.5
Dicalcium phosphate	13.0	11.7	11.7	11.7
Sodium chloride	3.0	-	-	-
Vitamin-mineral premix[1]	5.0	4.50	4.50	4.50
Rovabio® Excel AP	-	-	0.05	-
Alginate lyase	-	-	-	0.10

1- Vitamin-mineral premix, VitaTec®, provided by Tecadi, Santarém Portugal. Per 1 kg of premix: Vitamin A – 3,000,000 UI, Vitamin D3 – 500,000 UI, Vitamin E – 10,000 mg, Vitamin B1 – 500 mg, Vitamin B2 – 1,000 mg, Vitamin B6 – 500 mg, Vitamin B12 – 5 mg, Vitamin H2 – 18,75 mg, Vitamin K3 – 500 mg, Vitamin B5 – 3,750 mg, Vitamin B3 – 6,250 mg, Vitamin B9 – 62.5 mg, Choline chloride – 50,000 mg, Cu – 38,750 mg, Zn – 27,500 mg, Mn – 12,500 mg, I – 200 mg, Se – 50 mg, Fe – 25,000 mg, butyl-hydroxytoluene – 50 mg.
